# Neuronal Differentiation of Human Mesenchymal Stem Cells Using Exosomes Derived from Differentiating Neuronal Cells

**DOI:** 10.1371/journal.pone.0135111

**Published:** 2015-08-06

**Authors:** Yuji S. Takeda, Qiaobing Xu

**Affiliations:** Department of Biomedical Engineering, Tufts University, Medford, MA, United States of America; University of Torino, ITALY

## Abstract

Exosomes deliver functional proteins and genetic materials to neighboring cells, and have potential applications for tissue regeneration. One possible mechanism of exosome-promoted tissue regeneration is through the delivery of microRNA (miRNA). In this study, we hypothesized that exosomes derived from neuronal progenitor cells contain miRNAs that promote neuronal differentiation. We treated mesenchymal stem cells (MSCs) daily with exosomes derived from PC12 cells, a neuronal cell line, for 1 week. After the treatment with PC12-derived exosomes, MSCs developed neuron-like morphology, and gene and protein expressions of neuronal markers were upregulated. Microarray analysis showed that the expression of miR-125b, which is known to play a role in neuronal differentiation of stem cells, was much higher in PC12-derived exosomes than in exosomes from B16-F10 melanoma cells. These results suggest that the delivery of miRNAs contained in PC12-derived exosomes is a possible mechanism explaining the neuronal differentiation of MSC.

## Introduction

Spinal cord injuries severely affect motor functions and are currently the 2nd leading cause of paralysis in the United States. Following spinal injury, regeneration of the injured nerve is inhibited by glial scar formation, created by fibroblasts, neuroglia, monocytes, and endothelial cells [1, 2].

As technology improves, the treatment of spinal cord injuries through regenerative medicine is an increasingly promising approach [3]. The feasibility and safety of stem cell transplants have been clinically tested [4–6], and the use of mesenchymal stem cells (MSC) in particular has been extensively studied, as it is easier to obtain autologous MSCs than neural cells [7–9]. MSCs have been known to differentiate into neural cell types; for example, Prabhakaran et al. differentiated MSCs into neurons on a scaffold with a cocktail of induction agents [10]. Other groups have shown that both conditioned media from and coculture with neural cells (neurons, oligodendrocytes, and Schwann cells) induce differentiation of MSCs into neural cells [11–13]. These studies demonstrate that soluble components play a role in the differentiation of MSCs.

Exosomes are nanovesicles containing functional proteins and genetic materials, such as mRNA and microRNA (miRNA), which are secreted from many types of cells [14, 15]. Exosomes work as a vehicle for intercellular communication: in the nervous system, exosomes guide axonal development, modulate synaptic activity, and help regenerate peripheral nerve tissues [16]. Lopez-Verrilli et al. demonstrated neurite extension of dorsal root ganglion (DRG) *in vitro* and axonal regeneration *in vivo* after treatment with exosomes derived from primary Schwann cells [17]. One of the possible mechanisms through which exosomes promote neural regeneration is via miRNA contained in the exosomes. For example, Xin et al. reported that exosomes derived from MSC promote neurogenesis, neurite remodeling, and functional recovery after stroke. Furthermore, they demonstrated that the neurological recovery is promoted by the transfer of miR-133b from MSCs to neurons and astrocytes via MSC-derived exosomes [18, 19]. miRNA is also known to affect cell growth and direct differentiation of stem cells into many types of cells, including neurons [20, 21].

We hypothesize that exosomes derived from differentiating neuronal progenitor cells are enriched with miRNA that can induce neuronal differentiation of stem cells. In this study, we demonstrate that exosomes derived from PC12 cells, a neuronal cell line, can induce differentiation of human mesenchymal stem cells (hMSCs) into neuron-like cells. We furthermore propose a possible mechanism for the observed differentiation: the delivery of miRNAs from the exosome to the recipient hMSCs.

## Materials and Methods

### Exosome isolation

PC12 cells (ATCC, Manassas, VA) were cultured in Dulbecco’s modified Eagle’s medium (DMEM; Gibco, Grand Island, NY) supplemented with 10% horse serum (Gibco), 5% fetal bovine serum (FBS; Gibco), and 100 units/mL penicillin streptomycin (Pen Strep; Gibco). For exosome collection, PC12 cells were seeded at 5.7 × 10^2^ cells/cm^2^. Two days after seeding in the growth medium, the medium was changed to differentiation medium (DMEM supplemented with 2% horse serum, 100 ng/mL nerve growth factor (NGF; Sigma-Aldrich, St. Louis, MO), and 100 units/mL Pen Strep), with any exosomes contained in the serums used to create the media removed before use by ultracentrifugation (100000 × g [40000 rpm], overnight) [22]. The differentiation medium was then decanted and stored every 3 days as ‘conditioned media’. A Type 70 Ti rotor (Beckman Coulter, Brea, CA) was used for all ultracentrifugation processes.

Exosomes were then isolated from this conditioned medium by differential centrifugation [23]. First, cell debris was removed by centrifuging at 300 × g and 2000 × g for 20 min each. Microvesicles were then isolated by ultracentrifugation at 10000 × g (12000 rpm) for 45 min. The exosome-containing supernatant was filtered using a syringe filter with a pore size of 0.20 μm (Millipore, Billerica, MA). Finally, exosomes were collected by ultracentrifugation at 100000 × g (40000 rpm) for 150 min. The pellet of exosomes was resuspended in phosphate buffer saline (PBS; Fisher Scientific, Pittsburgh, PA) and stored at −80°C before use.

As a control, exosomes were collected from the conditioned medium of B16-F10 cells (ATCC), a melanoma cell line, using the same method as described above. B16-F10 cells were cultured in DMEM supplemented with 10% FBS and 100 units/mL Pen Strep.

The amount of exosomes collected was measured by determining protein concentration using Thermo Scientific Pierce BCA Protein Assay (Fisher Scientific).

### Morphological analyses of exosomes

The particle size of the exosomes was measured with dynamic light scattering (DLS) using a NanoBrook ZetaPALS (Brookhaven, Holtsville, NY). The exosomes were also observed with transmission electron microscopy (TEM; FEI, Hillsboro, Oregon).

### MSC culture

hMSCs were isolated from fresh bone marrow aspirate (Lonza, Allendale, NJ) as previously described [24]. hMSCs were expanded in DMEM, 10% FBS, 1% MEM Non-Essential Amino Acids Solution (Gibco), 100 units/mL Pen Strep, and 2 ng/mL basic fibroblast growth factor (bFGF) (Gibco). After expansion, hMSCs were seeded on culture plates (Falcon, Tewksbury, MA) at a seeding density of 3.0 × 10^3^ cells/cm^2^. The hMSCs were then cultured in DMEM, 10% FBS, 1% MEM Non-Essential Amino Acids Solution, 100 units/mL Pen Strep. Exosome (40 μg protein/mL medium) was added to the culture medium every day. The culture medium was changed every 3 days. All hMSCs used for this study were at passage 4.

### Immunostaining

hMSCs in culture were treated with exosomes for 7 days, after which microtubule-associated protein 2 (MAP2), 160 kDa neurofilament (NF160), and neuron-specific enolase (NSE) were immunostained as reported previously [25]. Briefly, the cells were first fixed in 10% buffered formalin (Fisher Scientific) and then permeabilized using 0.1% Triton X-100 (Electron Microscopy Sciences, Hatfield, PA). After blocking with 2% dry milk in PBS for 1 h, the samples were incubated with the primary anti-MAP2, NSE (Santa Cruz Biotechnology, Dallas, TX), and NF-160 (Sigma-Aldrich) antibodies. The excess antibody was removed by rinsing with PBS Tween-20, and then the samples were incubated with a secondary antibody conjugated with fluorescein isothiocyanate (FITC) (Sigma-Aldrich). Cell nuclei were counterstained using Fluoroshield with DAPI (Sigma-Aldrich). The images were taken with a fluorescent microscope (Keyence, Itasca, IL).

### Western blot

Following 7 days of exosome treatment, cells were removed from the culture plate using a cell scraper (Falcon) and lysed with cell lysis buffer containing 1% Triton X-100 and a protease inhibitor cocktail (Sigma-Aldrich). After determination of the protein concentration by BCA assay, the protein samples were denatured at 90°C for 5 min. The samples (30 μg) were loaded with LDS buffer (Life technologies) in a Bis-Tris gel (Life technologies). The gel was run in MES Buffer (Boston BioProducts, Ashland, MA) at 170 V for 45 min. The proteins were then transferred to a PVDF membrane (Life Technologies) at 30 V for 2 h in the transfer buffer (Boston BioProducts) with 20% methanol (BDH, Radnor, PA). After blocking with 5% dry milk in PBS, the membrane was incubated with primary antibodies against MAP2, NSE, and glyceraldehyde phosphate dehydrogenase (GAPDH) (Abcam, Cambridge, MA), and then the secondary antibody conjugated with horseradish peroxidase (HRP) (Life Technologies). The blots were detected with enhanced chemiluminescence (ECL) using an imager (Syngene, Frederick, MD). Western blotting of PC12 exosomes was performed using the same method described above.

### Quantitative PCR (qPCR) analysis

After 7 days of culture, total RNA was isolated from hMSCs using miRNeasy micro kit (Qiagen, Limburg, Netherlands), according to the manufacturer’s protocol. cDNA was synthesized from the RNA using a High-Capacity cDNA Reverse Transcription Kit (Life Technologies) and a thermal cycler (PTC-100, Bio-Rad, Hercules, CA). A qPCR assay was performed using LightCycler 480 SYBR Green I Master (Roche, Basel, Switzerland) and an Mx3000P (Agilent, Santa Clara, CA). The mRNA expression levels of MAP2 and NSE were normalized to that of GAPDH. The primers were designed to be specific to human, and do not match to rat genes. Sequences of the primers (Integrated DNA Technologies, Coralville, IA) are as follows: MAP2 forward primer, GGAACCAACTCTCTCTGGATTT; reverse primer, GCATTCTCTCTTCAGCCTTCT. NSE forward primer, CTGTATCGCCACATTGCTCAGC; reverse primer, AGCTTGTTGCCAGCATGAGAGC. GAPDH forward primer, ACCACAGTCCATGCCATCAC; reverse primer, TCCACCACCCTGTTGCTGTA.

The results of qPCR were analyzed using the method of Livak and Schmittgen [26]. The differences were statistically evaluated using one-way analysis of variance (ANOVA) and Dunnett's post-hoc test, which compared the values of exosome-treated samples with that of the non-treated sample. Each experiment was performed in triplicate.

### MicroRNA profiling

Exosomal RNA samples were isolated using a miRNeasy micro kit and submitted to Ocean Ridge Biosciences (Palm Beach Gardens, FL) for microRNA microarray processing. The DNA was digested with RNase free DNase I (Epicentre) and re-purified on Qiagen RNeasy Minelute columns (Qiagen). RNA (30 ng) for each sample was 3’-end labeled with Oyster-550 fluorescent dye using the Flash Tag RNA labeling Kit (Genisphere, Hatfield, PA). The labeled RNA samples were hybridized to the microRNA microarrays overnight according to conditions recommended by the manufacturer. The microarrays were scanned on an Axon Genepix 4000B scanner, and data was extracted from images using GenePix V4.1 software. The sequences of the probes are shown in S1 File.

Log 2 transformed data for the detectable rat and mouse probes were used as input for hierarchical clustering using Cluster 3.0 software [27]. Genes were median centered prior to hierarchical clustering. Hierarchical clustering was conducted using centered correlation as the similarity metric and average linkage as the clustering method.

## Results and Discussion

### Experimental design

**Fig 1:**
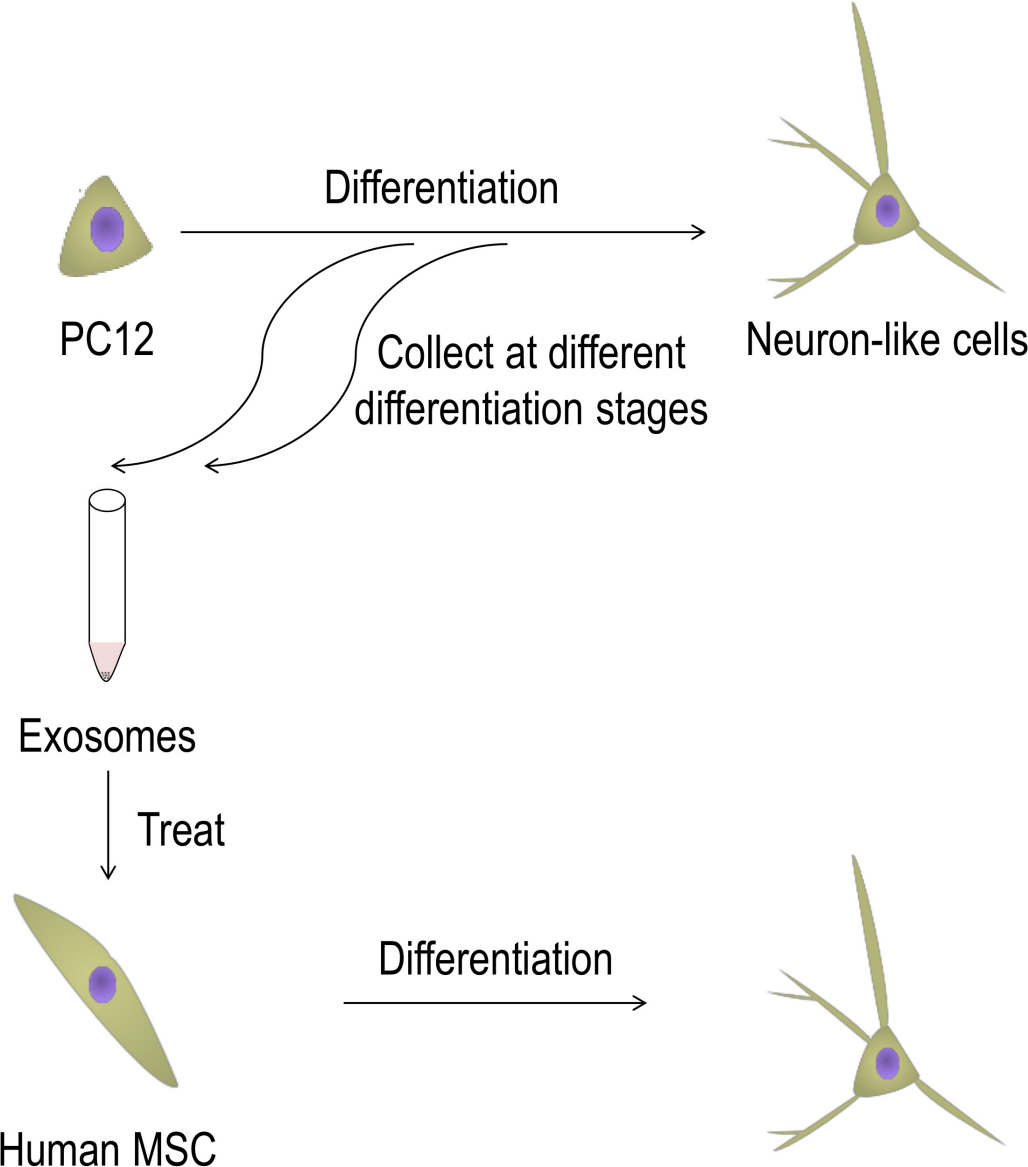
Schematic representation of this study. Exosomes were collected from the conditioned media of PC12 cells differentiating into neuron-like cells. Human mesenchymal stem cells (hMSC) were dosed with the exosomes, and then evaluated for differentiation.

Fig 1 illustrates the experimental design used to evaluate the potential of neuronal cell-derived exosomes to induce neuronal differentiation of hMSC. As a proof of concept, we collected exosomes from PC12 cells, a rat neuronal cell line, that differentiate into neuron-like cells following the stimulation with NGF in one week [28]. Briefly, exosomes were isolated by differential centrifugation of conditioned media from PC12 cells at various differentiation stages. We termed the exosome from undifferentiated PC12 cells before the NGF treatment “D0 exosome”, and the exosomes from PC12 cells treated with NGF for 3 and 9 days “D3” and “D9 exosome”, respectively. We also collected exosomes from B16-F10 cells, a melanoma cell line, as a control.

### Characterization of the exosomes

**Fig 2:**
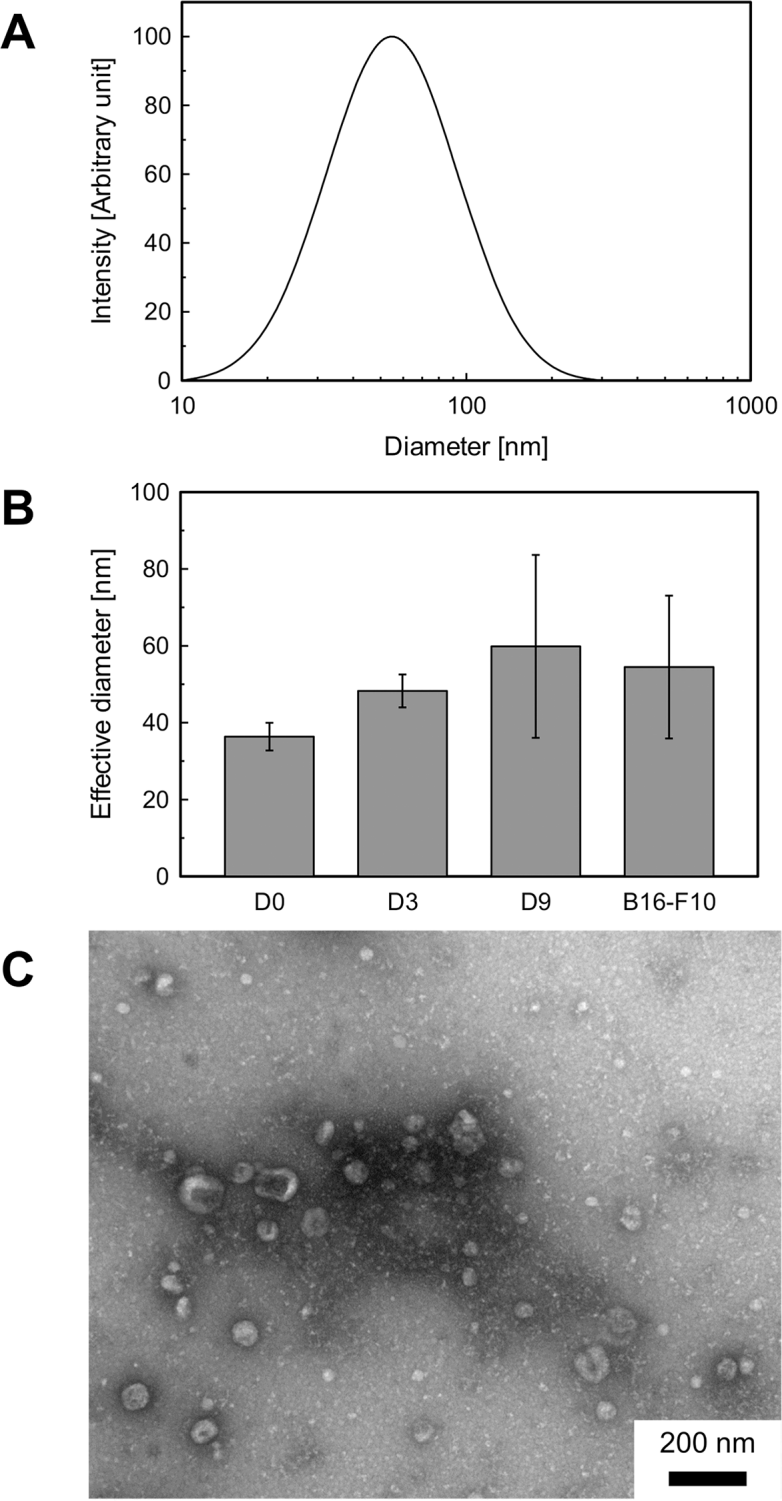
Size and morphology of exosomes. (A) Typical size distribution for an exosome sample derived from PC12 cells, measured with dynamic light scattering (DLS). (B) DLS analysis of exosome samples. *n* = 9. All error bars represent standard deviation. (C) Transmission electron microscopy (TEM) image of a typical exosome sample derived from B16-F10 cells. Scale bar, 100 nm.

The size of exosomes was evaluated using dynamic light scattering (DLS) (Fig 2A and 2B). The effective diameter of exosomes was found to be 30–85 nm, which is consistent with reports by other researchers (30–100 nm) [16, 18, 29]. There were no significant differences between the sizes of exosomes collected on different days of differentiation. The spherical morphology and size of the exosomes were confirmed with TEM (Fig 2C).

### Neuronal differentiation induced by exosomes

**Fig 3:**
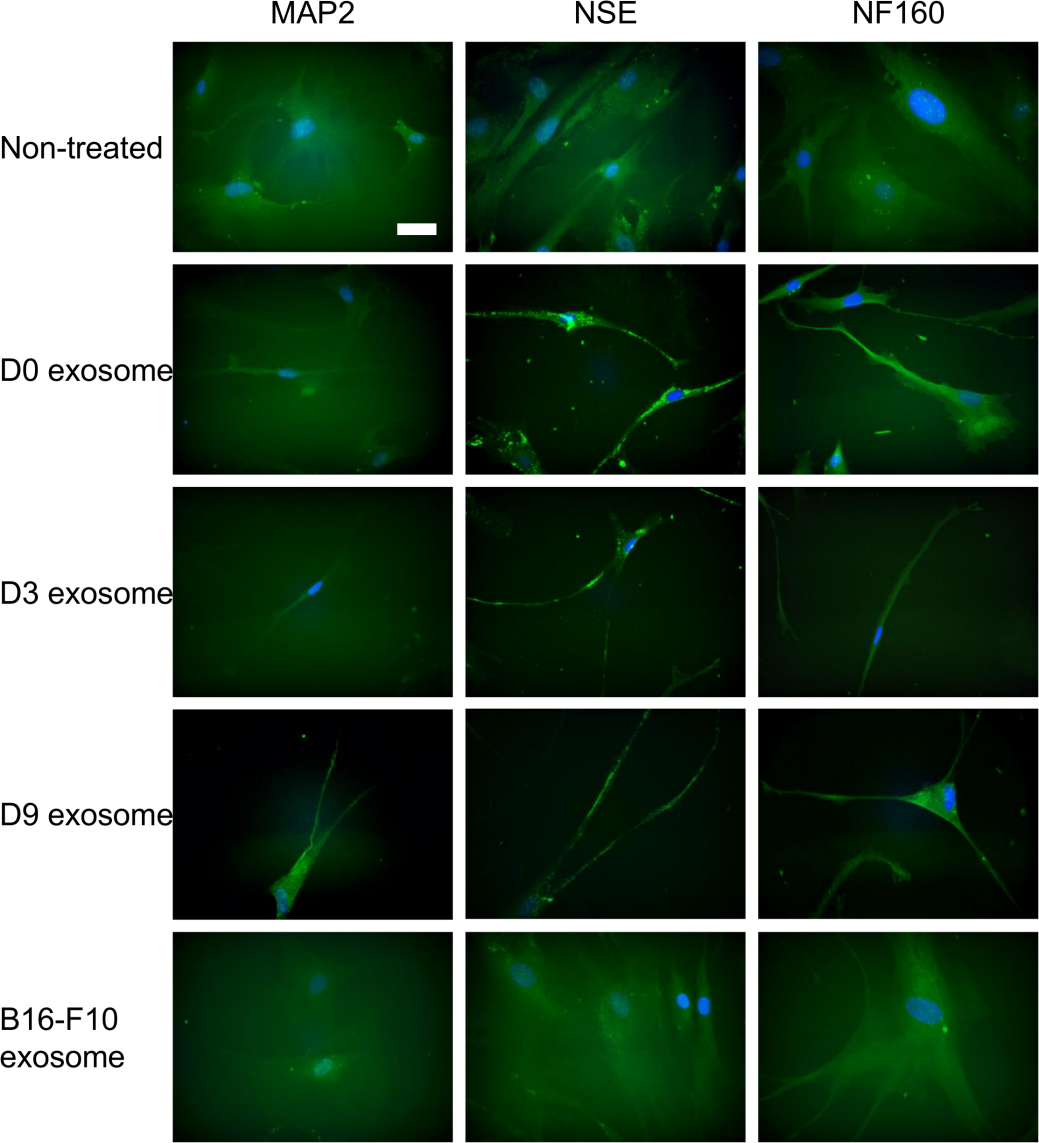
Cell morphology of hMSC after treatment with exosomes from neuronal cells. hMSCs were treated for 1 week with exosomes derived from differentiating PC12 cells. MAP2, NSE, and 160 kDa neurofilament (NF160) were immunostained. As negative controls, non-treated hMSCs and hMSCs treated with exosomes derived from B16-F10 cells are shown. Blue: cell nuclei. Scale bar, 50 μm.

hMSCs were treated daily with the exosomes for 1 week. The cell morphology of hMSCs was observed with immunofluorescence microscopy using an anti-MAP2, NSE, and NF160 antibodies. As shown in Fig 3, neurite-like extensions were observed in the cells treated with exosomes derived from PC12 cells, while non-treated cells and hMSCs treated with exosomes derived from B16-F10 cells did not change their morphology. Immunostaining, especially for NSE, was more pronounced after the treatment with PC12 exosomes, compared to non-treated hMSCs. Cells treated with D9 exosomes appeared more positive for MAP2.

**Fig 4:**
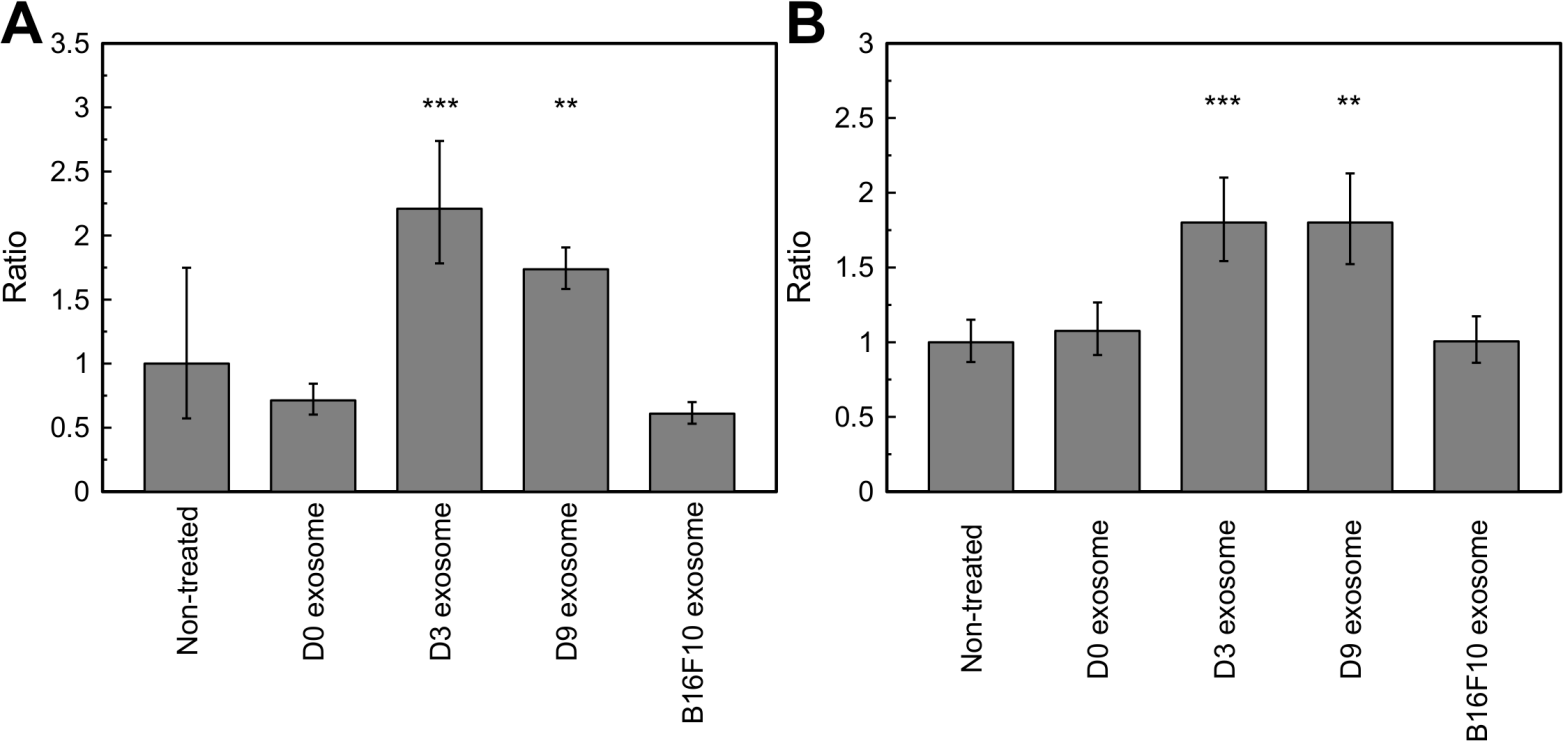
mRNA expression of hMSC treated with exosome samples for 1 week. (A) MAP2, (B) NSE. **: *p* < 0.01, ***: *p* < 0.001. Ratio compared with non-treated sample. All error bars represent standard deviation.

**Fig 5:**
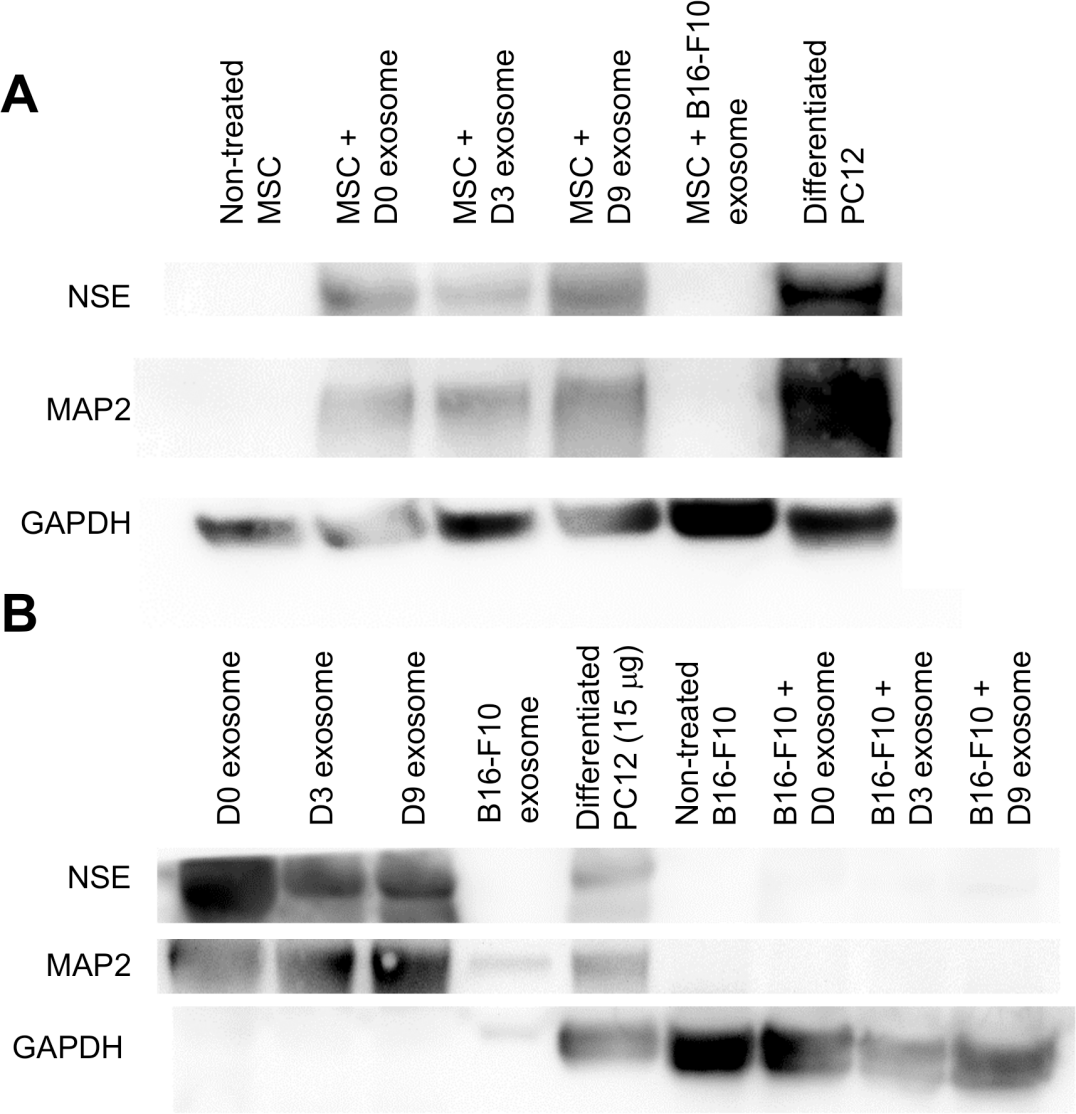
Western blot of neuronal marker proteins (NSE, MAP2, and GAPDH). (A) MSCs were treated with exosomes for 1 week. (B) PC12 exosomes and B16-F10 cells treated with PC12 exosomes. Cell lysate from differentiated PC12 cells, treated with NGF for 1 week, was used as a positive control.

mRNA expression of neuronal markers in hMSCs was measured with qPCR. Both mRNA coding for MAP2 and NSE were upregulated after the treatment with D3 and D9 exosomes while exosomes derived from B16-F10 cells and undifferentiated PC12 cells (D0 exosome) did not change the gene expression in hMSCs (Fig 4). Western blotting revealed that treatment with exosomes derived from PC12 cells (D0, D3, and D9 exosomes) increased the protein expressions of MAP2 and NSE, while non-treated MSCs and cells treated with B16-F10 exosome did not express detectable amount of these proteins (Fig 5A). Since the antibodies used for this experiment recognize both human and rat proteins, we examined whether protein transfer from exosomes to recipient hMSCs affected the result of Western blotting. Western blotting of exosomes derived from PC12 cells using the same antibodies detected these two proteins in exosomes derived from PC12 cells (Fig 5B, left). Exosomes derived from B16-F10 cells contained no or little amount of these proteins. In order to evaluate whether the transfer of these exosomal proteins to recipient cells leads to a positive signal of Western blot, we treated B16-F10 cells (MAP2 and NSE negative) with PC12 exosomes. As a result, MAP2 or NSE in the exosome-treated B16-F10 cells were not detected (Fig 5B, right). This result suggests that the volume of exosomal protein transfer was minimal and that the positive result of Western blot of exosome-treated hMSCs represents native expression in hMSCs. These results demonstrate that the exosomes derived from NGF-treated PC12 cells can promote the upregulation of neuronal markers.

### miRNA profiling in exosomes

**Fig 6:**
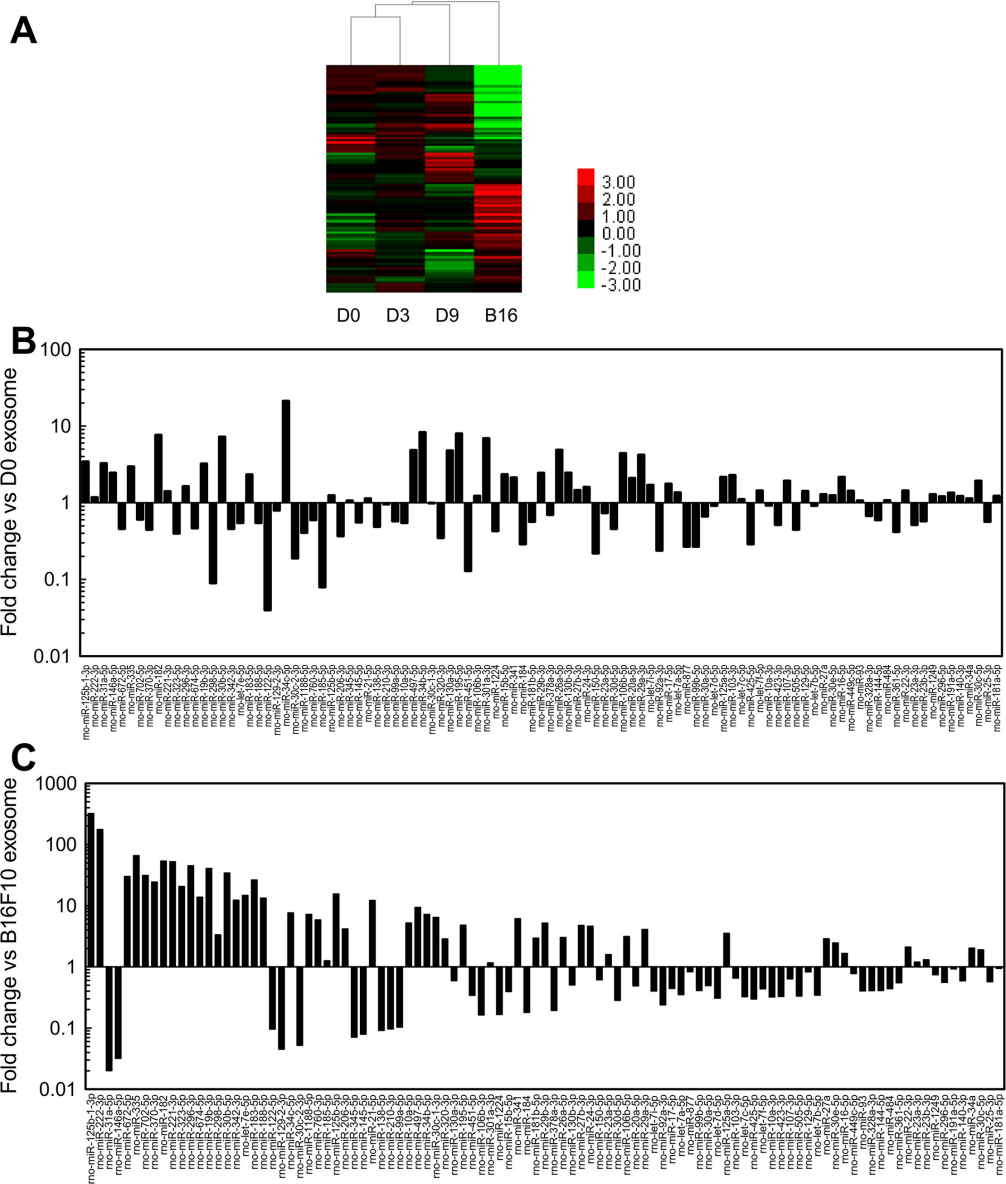
miRNA expression in exosomes. (A) 101 total probes common between rat and mouse species and detectable in at least 1 sample were used in analysis by hierarchical clustering. Intensities for the probes were log-2-transformed. (B, C) Comparison of miRNA expression between D9 and D0 exosome (B), and D9 and B16-F10 exosome (C).

In order to find out the possible mechanisms of the differentiation of hMSCs, we profiled miRNAs contained in the exosomes using microarray. We detected 101 miRNAs in the exosome samples (S1 File). Fig 6 shows the profile of miRNA expression in the exosome samples. Tables 1 and 2 are the lists of miRNAs enriched in PC12 exosomes. Nine miRNAs were enriched in PC12 exosome (> 2-fold higher expression than in B16-F10 exosome) and upregulated after the NGF treatment (Table 1). All 9 miRNAs in Table 1 are known to be enriched in neural tissues [30–32]. Notably, the expression of miR-125b was 319 times higher in D9 exosome than B16-F10 exosome, and the expression was upregulated after stimulation with NGF (Fig 6B and 6C, Table 1). miR-125b has been known to differentiate SH-SY5Y cells and MSCs into neuron-like cells [33, 34]. Another group demonstrated the upregulation of miR-125b during the differentiation of neural stem cells [35]. These reports suggest that delivery of miR-125b in the exosomes derived from differentiating PC12 cells is one of the possible mechanisms of neuronal differentiation of hMSC. On the other hand, miR-182 and 183 have been reported as becoming downregulated after peripheral nerve and spinal cord injury [31, 36]. Yu et al. reported the decrease of proliferation and migration of Schwann cells transfected with miR-182 [37]. The effects of these miRNAs on MSCs will be evaluated in detail in a future study.

**Table 1:** miRNAs enriched in PC12 exosomes and upregulated after NGF treatment.

Name of miRNA	Fold difference: D9 vs B16-F10	Fold difference: D9 vs D0	Possible roles of miRNA
**miR-125b**	319	3.41	Highly expressed in cortical neurons and spinal cord [30, 38]. Involved in differentiation into neuron [33–35].
**miR-335**	65.5	2.94	Highly expressed in cortical neurons [30].
**miR-182, 183**	53.3, 26.3	7.59, 2.32	Highly expressed in DRG and downregulated after peripheral and spinal nerve injury [31, 36]. Upregulated after sciatic nerve injury, inhibiting Schwann cell proliferation and migration [37].
**miR-19b**	40.4	3.21	Highly expressed in cortical neurons [30].
**miR-30b**	34.1	7.19	Highly expressed in cortical neurons and spinal cord [30, 38].
**miR-341**	6.10	2.11	Highly expressed in cortical neurons [30].
**miR-29b**	5.17	2.43	Upregulated after peripheral nerve injury [31].

**Table 2:** miRNAs enriched in PC12 exosomes but not upregulated after NGF treatment.

Name of miRNA	Fold difference: D9 vs B16-F10	Possible roles of miRNA
**miR-221, 222**	52.1, 176	Highly expressed in cortical neurons [30]. Promotes neurite outgrowth of DRG neurons [39], and proliferation and migration of Schwann cells [40]. Upregulated after spinal cord injury [38].
**miR-296**	44.8	Angiogenesis [41].
**miR-702**	31.0	Proliferation [42].
**miR-672**	29.8	Expressed in DRG and downregulated after entrapment neuropathy [43]. Upregulated after spinal cord injury [38].
**let-7e**	26.5	Highly expressed in cortical neurons and spinal cord [30, 38]. Neurogenesis [44].
**miR-370**	24.2	Suppresses tumor proliferation [45, 46].
**miR-323**	20.5	Downregulated after spinal cord injury [38].
**miR-674**	13.7	Upregulated after spinal cord injury, involved in apoptosis [38].
**miR-188**	13.3	Expressed in DRG and upregulated after sciatic nerve resection [47].
**miR-342**	12.3	Upregulated after neurodegeneration [48]
**miR-21**	12.1	Upregulated after spinal cord injury [38]. Promotes axon growth [49]. Improves neurological functions after traumatic brain injury by inhibiting apoptosis and promoting angiogenesis [50].
**miR-30c**	8.14	Expressed in cortical neurons [30].
**miR-1188**	7.17	Unknown.
**miR-760**	5.84	Induces cellular senescence [51].
**miR-27a, 27b**	2.87, 4.74	Highly expressed in spinal cord [38].
**miR-24**	4.60	Highly expressed in spinal cord [38]. Proliferation of glioma [52].
**miR-206**	4.14	Upregulated after spinal cord injury [38]. Stimulates regeneration of neuromuscular synapses [53]. Induces proliferation and apoptosis of neural cells [54].
**miR-181b**	2.94	Expressed in cortical neurons [30].

Some miRNAs were enriched in PC12 cells, but retained constant expression levels after the treatment with NGF (Table 2). Notably, the expressions of miR-221 and 222 were much higher in exosomes derived from PC12 cells than those from B16-F10 cells, (Fig 6C, Table 2). Gu et al. reported that miR-222 promotes neurite outgrowth of DRG neurons [39], and proliferation and migration of Schwann cells [40]. The existence of these miRNAs in the exosome could have played a role in the upregulated neuronal marker expression in hMSC.

Interestingly, not all miRNAs in Tables 1 and 2 have been reported in the context of nerve tissues. Only a few reports are available for some of the miRNAs (such as miR-1188), which could have also induced differentiation. In addition, the research on the effects of miRNAs often focused on the effect of single miRNAs, while little is known about interaction of multiple miRNAs. A bioinformatics-focused approach could reveal the synergistic interaction of miRNAs for nerve regeneration [55, 56]. Future study will evaluate the effects of these unknown miRNAs as well as the combination of multiple miRNAs on nerve regeneration.

## Conclusion

In this study, we showed that exosomes derived from neuron progenitor cells at various differentiation stages can differentiate hMSC into neuron-like cells. We also demonstrated that the exosomes contain miRNAs known to play a role in neuronal differentiation. The differentiation could be caused by the delivery of the mixture of miRNA, mRNA, and proteins contained in the exosome, and the exact mechanism remains to be determined in future studies.

## Supporting Information

S1 File miRNA microarray of exosome samples. This file contains the sequences of the probe used for the microarray assay. The raw data of the microarray was also contained.
(XLSX)
